# ﻿*Chrysanthemumdabieshanense*, a new name for ﻿*Chrysanthemumvestitum* var. ﻿*latifolium* (Asteraceae, Anthemideae)

**DOI:** 10.3897/phytokeys.202.80554

**Published:** 2022-07-21

**Authors:** Zhixi Fu, Xiaofeng Liu, Aiguo Zhen, Xinxin Zhu, Kamil Konowalik, Yueping Ma, Pan Li

**Affiliations:** 1 College of Life Sciences, Sichuan Normal University, Chengdu 610101 China; 2 Sustainable Development Research Center of Resources and Environment of Western Sichuan, Sichuan Normal University, Chengdu 610101, China; 3 Foresty Bureau of Yingshan County, Huanggang 421124 China; 4 College of Life Sciences, Xinyang Normal University, Xinyang 464000, Henan, China; 5 Department of Plant Biology, Institute of Environmental Biology, Wrocław University of Environmental and Life Sciences, Wrocław 5b, 51-631, Poland; 6 College of Life and Health Sciences, Northeastern University, Shenyang 110004, China; 7 College of Life Sciences, Zhejiang University, Hangzhou 310058 China

**Keywords:** Asteraceae, China, *Chrysanthemum* endemism, taxonomy

## Abstract

Recent phylogenetic analyses have revealed that Chrysanthemumvestitumvar.latifolium and C.vestitumvar.vestitum were placed in different clades based on their chloroplast genomes and nuclear LFAFY gene sequences. Accordingly, based on previous morphological analysis, molecular phylogenetic results, fieldwork, and herbarium studies, Chrysanthemumvestitumvar.latifolium should be raised to the species level. Considering the condition of the material found and Articles 6.9, 6.11, 41.2, 58.1 of the International Code of Nomenclature for Algae, Fungi, and Plants (*Shenzhen Code*) that is currently in force, *Chrysanthemumdabieshanense* Z.X.Fu, A.G.Zhen, & Y.P.Ma, **nom. nov.** is proposed as the new name for Chrysanthemumvestitumvar.latifolium J.Zhou & Jun Y.Chen. The detailed emended description, distribution map, insights into its habitat, and an updated comparative morphological study are presented in this study.

## ﻿Introduction

*Chrysanthemum* L. is a genus of the tribe Anthemideae that contains approximately 40 species. This genus is mainly distributed in temperate Asia (Oberprieler et al. 2007), with approximately 23 species in China (Shih et al. 2011; Meng et al. 2020). The genus is characterized by subshrubs or perennial herbs with pinnately or palmately divided alternate leaves, female ray florets, white or red apical appendages of anthers, and faintly 5–8-ribbed achenes (Shih et al. 2011). Furthermore, recent molecular phylogenetic studies have demonstrated that Chinese *Chrysanthemum* should be divided into two groups: the *Chrysanthemumzawadskii* group, which is distributed in northern China and has erect stems and large capitula with white or purple ray florets, and the *Chrysanthemumindicum* group, which is distributed from north to south China and has creeping stems and capitula with yellow or white ray florets (Liu et al. 2012; Li et al. 2014; Ma et al. 2020).

According to the phylogenetic study of chloroplast genomes and the nuclear *LFAFY* gene by Ma et al. (2020), Chrysanthemumvestitumvar.vestitum from the Funiu Mountain in Henan Province and C.vestitumvar.latifolium from the Tianzhu Mountain in Anhui Province represent two distinct clades. Morphologically, C.vestitumvar.latifolium is not similar to other species of Clade I (Shih et al. 2011). Accordingly, based on the morphological and molecular results, we propose that the variety Chrysanthemumvestitumvar.latifolium should be raised to the species level.

This evidence of phylogenetic results seems to be sufficient for a new taxonomic decision. This study aimed to describe one of the species of *Chrysanthemum* and investigate its phylogenetic affinities based on molecular and morphological data. Combined with previous morphological and field studies, we also provide a distribution map and information on the taxonomy of *Chrysanthemumdabieshanense*.

## ﻿Materials and methods

**Figure 1.**
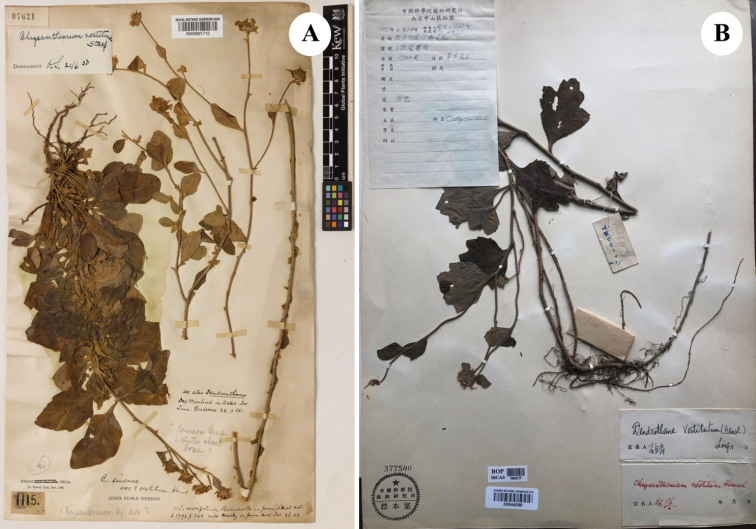
The images of the holotype of *Chrysanthemumvestitum* and Chrysanthemumvestitum(Hemsl.)Stapf.var.latifolium**A** the lectotype of *Chrysanthemumvestitum* (Hemsl.) Stapf. Image courtesy of the Royal Botanic Gardens, Kew (K). (Type: China. Hubei: Yichang city, “Ichang and immediate neighborhood”, Sep 1886, *A. Henry 1115* lectotype, K000891712) **B** the holotype of Chrysanthemumvestitum(Hemsl.)Stapf.var.latifolium J.Zhou et J.Y.Chen. Image courtesy of the Herbarium PE, (Type: China, Anhui Province: Yuexi County, Baojia River, alt. 1500 m, 24 Sep 1953, *East China Station Inst. Bot. 6935* holotype, PE 00544099!).

**Figure 2.**
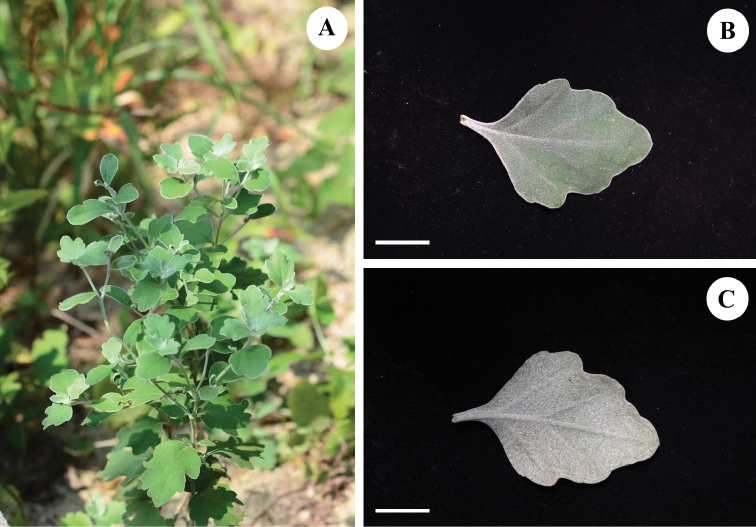
A plant of Chrysanthemumvestitum(Hemsl.)Stapfvar.latifolium collected at Tianzhu Mountain, Dabieshan mountains (The voucher no. MYP-20160826, WUK, Ma et al. 2020) **A** plant growing in natural habitat **B** the adaxial side of leaf **C** the abaxial side of leaf. Scale bar: 2 cm (**B, C**). Photographed by Yue-ping Ma.

We employed standard techniques for morphological studies of herbarium specimens and digital images of the most closely related species from the herbaria CSH, HIB, K, KUN, PE, WUK; acronyms follow Thiers (2022), including the holotype specimens of *Chrysanthemumvestitum* (Hemsl.) Stapf (Fig. 1A) (K, images seen). Dr Z.X. Fu visited the PE herbarium (Institute of Botany, Chinese Academy of Sciences) in June 2021 and compared and checked the holotype specimen of Chrysanthemumvestitum(Hemsl.)Stapf.var.latifolium J.Zhou et J.Y.Chen (East China Station Inst. Bot. 6935, Fig. 1B, PE). We also verified that morphological characteristics of voucher specimen MYP-20160826 (Fig. 2, Ma et al. 2020, photo from Y.P. Ma) are identical to those of the type specimen of C.vestitumvar.latifolium.

The morphological characteristics of C.vestitumvar.latifolium and its related species were examined for comparative research based on measurements of herbarium specimens (Table 1), supplemented by photos of mature living plants collected from the field (photos from A.G. Zhen and X.X. Zhu). The localities were sorted according to county-level administrative divisions of the People’s Republic of China.

## ﻿Results and discussion

Zhou and Chen (2010) reported a new variety: Chrysanthemumvestitum(Hemsl.)Stapf.var.latifolium J.Zhou et J.Y.Chen from China (Fig. 1B). In their study, the holotype specimen of Chrysanthemumvestitumvar.latifolium was collected from the Tianzhu Mountain (Anhui Province, Fig. 1B). The variety Chrysanthemumvestitumvar.latifolium is mainly distributed on the western slopes of the Dabie Mountains, throughout the Anhui and Hubei provinces (Qi et al. 2021). It has long been treated as a variety of *Chrysanthemumvestitum* (Fig. 1A). Based on a recent study by Meng et al. (2020), principal component analysis of leaf length (L), leaf width (W), petiole length (P), and relative petiole length (B = P/L) between *C.vestitum* and C.vestitumvar.latifolium was conducted, and the two varieties of *C.vestitum* differed slightly. The difference between the new variety and the original species is that the new variety is less branched and has orbicular and ovoid leaves that are 4–7 cm long and 3–5 cm wide, and the diameter of its capitula is larger, reaching 4.5–5.0 cm (Table 1). The morphology of C.vestitumvar.latifolium is distinct from other species in the same clade in terms of the leaf shape and length of capitula (Shih et al. 2011; Ma et al. 2020) (Table 1).

**Table 1.** Comparative measurements in *Chrysanthemumdabieshanense* (= C.vestitumvar.latifolium) and its related species (based on Shih et al. 2011 and additional specimens at the herbaria visited).

	*C.dabieshanense*	C.vestitumvar.vestitum	*C.mongolicum* (Clade I)	*C.indicum* (Clade I)	*C.zawadskii* (Clade I)	*C.lavandulifolium* (Clade I)
Leaf blades	orbicular or ovate-orbicular, 4–7 × 3–5 cm, obtusely 2- or 3-lobed	ovate, broadly ovate, oblong, 3.5–7 cm × 2–4 cm, margin repand-dentate	broadly ovate or elliptic 1–2 cm × 1.5–1.8 cm, bipinnatisect	ovate, long ovate, or elliptic-ovate,3–7 cm × 2–4m, bipinnatisect	ovate, broadly ovate, 1.4–4 ×1–3.5 cm, bipinnatisect	ovate, broadly ovate, elliptic-ovate, narrowly elliptic, 2–7 × 1.5–4.5 cm, bipinnatisect
Phyllaries	4 rows	4 rows	5 rows	5 rows	4 rows	5 rows
Capitula	4.5–5 cm in dia.	2–3 cm in diam.	3–4.5 cm in diam.	2.5–4 cm in diam.	1.5–4.5 cm in diam.	1–1.5 cm in diam.

**Figure 3.**
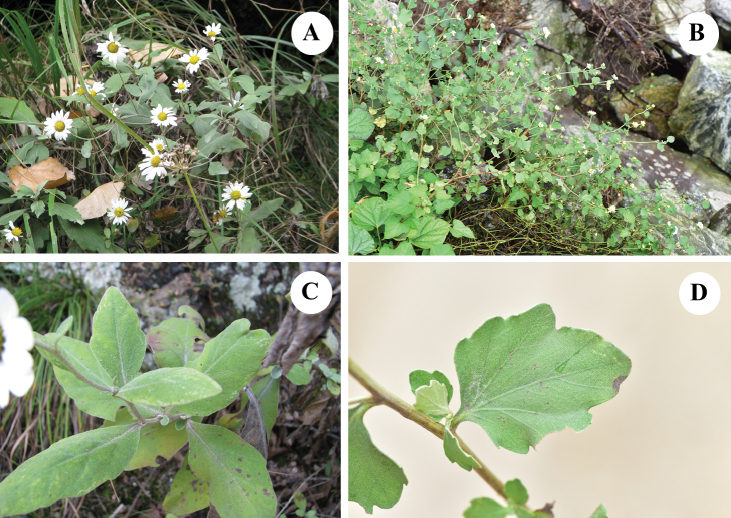
The images of *Chrysanthemumvestitum* and *Chrysanthemumdabieshanense***A, B** plants growing in natural habitat **C, D** adaxial side of leaf (**A, C***Chrysanthemumvestitum*, voucher *Z.X. Fu 610*, PE, Lushi county, Henan province, China, Photographs by Zhixi Fu). (**B, D***Chrysanthemumdabieshanense*, voucher *X.X.Zhu 089* CSH, Yingshan county, Hubei province, China. Photographs by Xinxin Zhu).

Based on a phylogenetic study of whole chloroplast genomes (Ma et al. 2020), two distinct clades were recognized in the genus *Chrysanthemum*. Clade I comprised *C.chanetii*, *C.indicum*, *C.lavandulifolium*, *C.nankingense*, *C.zawadskii*, *C.dichrum*, *C.mongolicum*, *C.oreastrum*, *C.glabriusculum*, *C.boreale*, *and C.vestitum*var.latifolium from Tianzhu Mountain in the Dabieshan Mountain area (the *Chrysanthemumzawadskii* group). Clade II consisted of *C.rhombifolium*, C.indicumvar.aromaticum, *C.potentilloides*, *C.hypargyrum*, *C.argyrophyllum*, and C.vestitumvar.vestitum (Fig. 3 A, C) (the *Chrysanthemumindicum* group). Based on the nuclear *LFAFY* gene, Chrysanthemumvestitumvar.latifolium and C.vestitumvar.vestitum were treated as two distinct species in different clades (Ma et al. 2020). Accordingly, based on the morphology and molecular results, we propose that the variety Chrysanthemumvestitumvar.latifolium should be raised to the species level.

### ﻿Taxonomic treatment

#### ﻿ Chrysanthemum dabieshanense

Taxon classificationPlantaeAsteralesAsteraceae

Z.X.Fu, A.G.Zhen, & Y.P.Ma nom. nov.

0332E47C-C741-58DD-BC5D-FA65340C0963

urn:lsid:ipni.org:names:77302160-1

≡ Chrysanthemumvestitumvar.latifolium J.Zhou & J.Y.Chen, Bull. Bot. Res. Harbin. 30: 649. 2010.

##### Note.

According to International Code of Nomenclature for algae, fungi, and plants (ICN) Articles 6.9, 6.11, 41.2, and 58.1 (Turland et al. 2018), *Chrysanthemumdabieshanense* Z.X.Fu, A.G.Zhen, & Y.P.Ma, nom. nov. is proposed here as an explicit substitute for the legitimate name Chrysanthemumvestitumvar.latifolium, because the epithet *latifolium* cannot be used for the present combination because of the existence of the name *Chrysanthemumlatifolium* (DC.) Baksay, Ann. Hist. Nat. Mus. Natl. Hung. 8: 161, 1957 (online resource from https://www.tropicos.org/name/50268974). The specific epithet “*dabieshanense*” refers to the name of the Dabieshan (= Ta-pieh) mountain area, located at the border of the Anhui, Hubei, and Henan Provinces, where the species occurs. *Chrysanthemumdabieshanense* is endemic and restricted to the Dabieshan Mountain area (Hubei and Anhui provinces). Therefore, we accept *Chrysanthemumdabieshanense* as a replacement name for Chrysanthemumvestitumvar.latifolium. A taxonomic treatment is presented.

##### Type.

China. Anhui Prov. Yuexi County, Baojia River, shady slope at the top of the hill, alt. 1500 m, 24 Sep 1953, East China Station Inst. Bot. 6935 (holotype, PE 00544099!, isotype NAS 00486826 photo seen) (Fig. 1B)

##### Description.

Perennial rhizomatous herbs, 60–100 cm tall. Stems sprawling, not much branched. Lower stem leaves withered at anthesis. Middle stem leaf blades orbicular or ovate-orbicular, 4–7 cm × 3–5 cm, grayish-green adaxially, grayish-white abaxially, margin above middle obtusely repand-dentate, distal stem leaves sessile or subsessile, capitula 3–10, 4.5–5 cm in diameter. Involucres cup-shaped; phyllaries in 4 rows, abaxially densely pubescent, scarious margin brown, outer phyllaries triangular or triangular-ovate, 3.5–4.5 mm, middle phyllaries lanceolate-ovate, ca. 6.5 mm, inner phyllaries obovate or oblanceolate-elliptic, 6–7 mm long. Ray floret lamina white, 1.2–2 cm long. Achenes ca. 1.5 mm long (Fig. 2, Fig. 3 B, D).

##### Specimens examined.

China – Anhui Prov. Jinzhai County, [without exact locality], *M.B. Deng & H.T. Wei 81350, 81196* (NAS); ibidem, *B. Chen CB07550* (CSH), Qianshan City, *S. J. Yang et al. 7193* (NAS), Y.P. Ma *MYP-20160826* (WUK); Hubei Prov. Yingshan County, Wujiashan Forest Farm, *X.X. Zhu 089* (KUN, CSH); Luotian County, Tiantangzai, *A.G. Zhen DBSYS708* (HIB); *X.X. Zhu 211858* (KUN).

##### Distribution and habitat.

**Figure 4.**
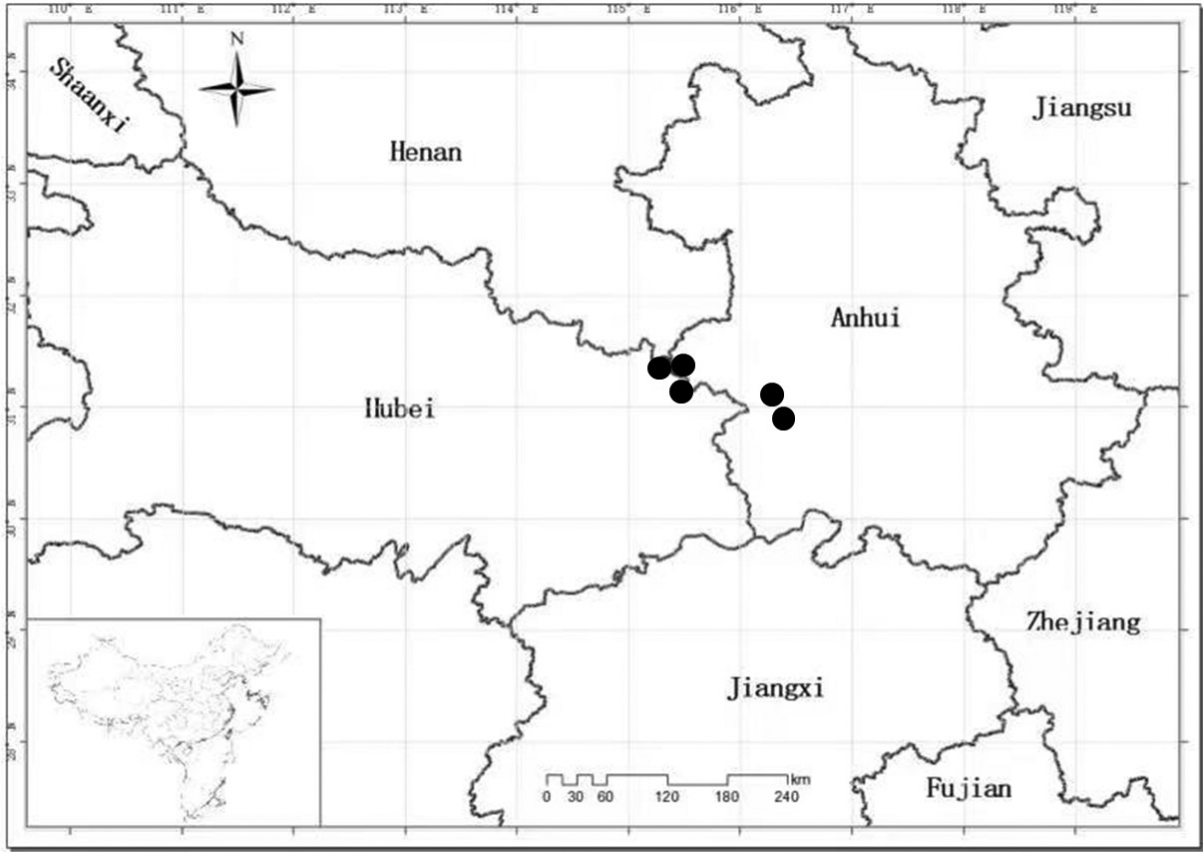
Distribution map of *Chrysanthemumdabieshanense* (black circles) in Anhui and Hubei provinces, China, based on the voucher specimen information.

Endemic to the Dabieshan mountain area (Anhui and Hubei Provinces, China, Fig. 4). It grows on shaded slopes, hills, and streamsides, at alt. 800–1600 m.

##### Phenology.

Flowering and fruiting are observed in October.

##### Chinese name.

Da-Bie-Shan-Ju (大别山菊).

## Supplementary Material

XML Treatment for Chrysanthemum dabieshanense
